# Pigment Composition of Nine Brown Algae from the Iberian Northwestern Coastline: Influence of the Extraction Solvent

**DOI:** 10.3390/md20020113

**Published:** 2022-01-31

**Authors:** Pascual Garcia-Perez, Catarina Lourenço-Lopes, Aurora Silva, Antia G. Pereira, Maria Fraga-Corral, Chao Zhao, Jianbo Xiao, Jesus Simal-Gandara, Miguel A. Prieto

**Affiliations:** 1 Nutrition and Bromatology Group, Analytical and Food Chemistry Department, Faculty of Food Science and Technology, Universidade de Vigo, Ourense Campus, E-32004 Ourense, Spain; pasgarcia@uvigo.es (P.G.-P.); c.lopes@uvigo.es (C.L.-L.); mass@isep.ipp.pt (A.S.); antia.gonzalez.pereira@uvigo.es (A.G.P.); mfraga@uvigo.es (M.F.-C.); jianboxiao@uvigo.es (J.X.); 2 Department for Sustainable Food Process, Università Cattolica Del Sacro Cuore, Via Emilia Parmense 84, 29122 Piacenza, Italy; 3 REQUIMTE/LAQV, Instituto Superior de Engenharia do Porto, Instituto Politécnico do Porto, Rua Dr António Bernardino de Almeida 431, 4200-072 Porto, Portugal; 4 Centro de Investigação de Montanha (CIMO), Instituto Politécnico de Bragança, Campus de Santa Apolonia, 5300-253 Bragança, Portugal; 5 Engineering Research Centre of Fujian-Taiwan Special Marine Food Processing and Nutrition, Ministry of Education, Fuzhou 350002, China; zhchao@live.cn; 6 College of Food Science, Fujian Agriculture and Forestry University, Fuzhou 350002, China; 7 Key Laboratory of Marine Biotechnology of Fujian Province, Institute of Oceanology, Fujian Agriculture and Forestry University, Fuzhou 350002, China; 8 International Research Center for Food Nutrition and Safety, Jiangsu University, Zhenjiang 212013, China

**Keywords:** solid–liquid extraction, Phaeophyceae, chlorophylls, carotenes, xanthophylls, bioactive natural products

## Abstract

Brown algae are ubiquitously distributed in the NW coastline of the Iberian Peninsula, where they stand as an underexploited resource. In this study, five solvents were applied to the extraction of pigments from nine brown algae, followed by their determination and quantification by HPLC-DAD. A total of 13 compounds were detected: Six were identified as chlorophylls, six were classified as xanthophylls, and one compound was reported as a carotene. Fucoxanthin was reported in all extracts, which is the most prominent pigment of these algae. Among them, *L. saccharina* and *U. pinnatifida* present the highest concentration of fucoxanthin (4.5–4.7 mg∙g^−1^ dry weight). Ethanol and acetone were revealed as the most efficient solvents for the extraction of pigments, showing a maximal value of 11.9 mg of total pigments per gram of dry alga obtained from the ethanolic extracts of *H. elongata*, followed by the acetonic extracts of *L. ochroleuca*. Indeed, ethanol was also revealed as the most efficient solvent according to its high extraction yield along all species evaluated. Our results supply insights into the pigment composition of brown algae, opening new perspectives on their commercial exploitation by food, pharmaceutical, and cosmeceutical industries.

## 1. Introduction

The current consumer demand in terms of product naturalness has forced the evolution of market preferences towards the exploitation of resources of biological origins [1]. While plants have been thoroughly employed for multiple applications by a wide range of industrial sectors, seaweeds are still considered an underexploited natural resource in Western countries [2]. In this regard, greater efforts are underway by facing the introduction of algal products in these countries, based on their reported health benefits, wellness and gastronomic properties [3]. Such bioactive features have raised the interest in the exploitation of algae in economically important sectors, as it is the case of food, pharmaceutical and cosmetic industries [4]. Among them, pigments have achieved much interest in algal research since they constitute a characteristic feature involved in the taxonomic classification and identification of these marine organisms. Indeed, three major families of seaweeds are defined according to their prominent coloration: green, red and brown seaweeds, which corresponding to three different phyla Chlorophyta, Rhodophyta and Ochrophyta, respectively. As a matter of fact, the chemical composition of seaweeds is highly different among phyla, especially pigments, but there are additional factors that play a significant role in the production of these compounds, such as seasonality, geographical area of harvesting and other environmental factors [5,6].

With respect to algal pigments, it is worth highlighting three different groups: carotenoids, including both carotenes and xanthophylls, chlorophylls and phycobilins [7,8,9]. In particular, brown seaweeds exhibit a rich composition of pigments of different biosynthetic origins, providing their characteristic pigmentation: (a) chlorophylls a and c; (b) carotenes, mainly represented by α-carotene and β-carotene; and (c) xanthophylls, considered as the most prevalent family of pigments in these species, including neoxanthins A and B, fucoxanthin and violaxanthin as the major compounds [10,11]. Thus, different combinations of these pigments are responsible for the natural pigmentation of algae, for instance, it is important to note that fucoxanthin and chlorophyll c are predominantly reported in the Phaeophyceae family in which brown algae belong [12,13].

Concerning their functionality, brown seaweed pigments have been predominantly used at an industrial level as coloring agents of food products and beverages and as animal feed to improve the appearance of pet foods and dairy products, such as poultry, fish and seafood [14]. Moreover, in addition to their roles as colorants, some pigments have also been reported to exhibit additional properties as health-enhancing compounds, showing a beneficial impact on human chronic disorders, such as diabetes, obesity, cancer, cardiovascular, inflammatory, neurodegenerative and immune diseases, as it was determined for fucoxanthin, zeaxanthin and astaxanthin [14]. In addition to pigments, the economical and biological value of brown algae is also supported by the production of fucoidan, the major polysaccharide found in these species, which has been recently recognized as a promising phytoconstituent thanks to its associated gastronomical, pharmaceutical and bioactive properties [15,16,17]. In this sense, the re-valorization of brown seaweed pigments is a promising strategy to incorporate these underexploited natural resources into a myriad of applications in different sectors. This work aims at the optimization of pigment extraction of different brown algae found in a great extent along the coastal Northwestern region of the Iberian Peninsula. In particular, nine brown algae, namely *Ascophyllum nodosum* (AN), *Bifurcaria bifurcata* (BB), *Fucus spiralis* (FS), *Himanthalia elongata* (HE), *Laminaria ochroleuca* (LO), *Laminaria saccharina* (LS), *Pelvetia* *canaliculata* (PC), *Sargassum muticum* (SM) and *Undaria pinnatifida* (UP), were subjected to pigment extraction. To assess an efficient extraction of these compounds at a laboratory scale, five solvents of different polarities, ranging from highly polar, such as ethanol, to poorly polar solvents, such as hexane, were selected to simultaneously figure out their influence on the isolation of different algal pigments. Overall, the achievement of optimized protocols will supply insights into the chemical characterization of underexploited natural sources in terms of pigment composition in brown algae and further help their valorization facing their industrial exploitation in important sectors, as it is the case of food and cosmeceutical industries.

## 2. Results and Discussion

The optimization of the extraction procedure is a crucial step when valorizing unexploited resources, as it happens to some of the brown algae involved in this study. As a result, such characterization is needed to supply insight into the potential applications of these marine organisms in different industrial sectors as promising biological producers of pigments. Indeed, some of the species selected for this study are classified as Generally Recognized As Safe (GRAS) by the US Food and Drug Administration (FDA), such as LS and UP [18], which facilitates their commercial exploitation for food purposes. Moreover, AN, FS, HE and SL are found in the list of brown algae under regulation for human food application by the European Union [19].

The results for the extraction yield are shown in Table 1. As reported in all species, the greatest yields were obtained for ethanolic extracts, and UP is the seaweed showing the highest value, 38.8%, followed by BB and HE, ranging 24–27%, respectively (Table 1).

The highest results found for UP when using ethanol could be motivated by the rich composition in terms of polar constituents of this species, containing 50.4% of polysaccharides, 19.7% of proteins and 9.2% of minerals, only showing a negligible 3.3% corresponding to lipids [20]. Compared with other solvents, extraction yields decreased while decreasing the polarity of solvents, thus reporting the lowest values in the cases of ethyl acetate and hexane-based extracts. These results make sense since the proportion of hydrophobic constituents in brown algae is quite low; hence, the efficiency of less polar solvents was already expected to be lower, as proved by the present experimental data. In consequence, these results show that ethanol is a very efficient solvent to be used in the extraction of algal samples, as it can dissolve a heterogeneous range of chemical components. In this sense, ethanol has been reported to efficiently extract polar compounds, such as proteins and derived molecules, as well as acting as a precipitating agent of polysaccharides, which constitute the highest proportion of chemical constituents in algae [15,16,17,21]. Indeed, the precipitating ability of ethanol is very convenient to avoid the presence of interferences during its analytical determination.

**Figure 1:**
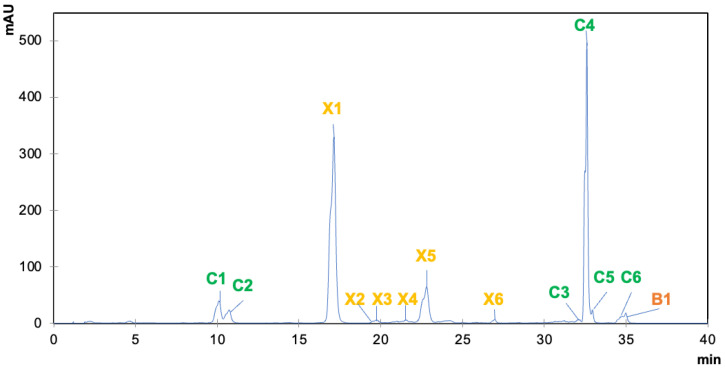
Total chromatogram of brown algae pigments and UV-Vis spectra of the compounds detected by HPLC-DAD. Compounds: C1, chlorophyll c2; C2, chlorophyll c1; C3, chlorophyll a isomer; C4, chlorophyll a; C5, chlorophyll a isomer; C6, pheophorbide A; X1, fucoxanthin; X2, violaxanthin; X3, auroxanthin; X4, fucoxanthin derivative; X5, Dihydrolutein; X6, Zeaxanthin; B1, β-carotene.

The pigment composition of brown algae, according to HPLC-DAD analysis of algal extracts indicated the presence of 13 different molecules (Figure 1). Among them, three families of pigments were recorded: chlorophylls, represented by six compounds; xanthophylls, also including six compounds; and carotenes, exclusively represented by β-carotene. Concerning chlorophylls, two families were reported in brown algae: chlorophylls a (Chl a) and c (Chl c), identified as compounds C1–C6. The identification of Chl c was based on the UV-Vis spectra of the detected compounds, since they present a wide Soret band absorption peak around 450 nm, together with a small peak at ~630 nm [22]. Moreover, the differentiation between different Chl c compounds is due to their structure, as Chl c_2_ and Chl c_3_ contain an extra double bond within their structural conjugated system that causes light absorption at larger wavelengths than Chl c_1_ [22]. For that reason, C1 could be differentiated from C2, the latter being putatively identified as Chl c_1_, since it presents peaks at shorter wavelengths (448, 580 and 632 nm) in comparison with C2 (452, 584 and 634 nm) (Figure 1, Table 1). Additionally, C2 may be identified according to the distribution of Chl c_2_ and c_3_ as constituents of biological organisms, being likely identified as Chl c_2_ since Chl c_3_ has been described almost exclusively in haptophytes rather than in diatoms [22]. Consequently, C1 was identified as Chl c_2_. The rest of the chlorophyll compounds (C3–C6) were related to the Chl a family because of the presence of both characteristic red band at 664 nm and Soret band at 432 nm [23]. Indeed, C4 was identified as Chl a in accordance with the standard used for quantification, whereas C3 and C5 compounds were considered as isomers of this pigment, with no significant changes in their absorption properties [23]. In the case of C5, it was identified as Chl a isomer, as previously reported by Zeb et al. [24] with a similar spectrum to that of Chl a, although a distinctive identification between Chl a epimer and anomer was not possible to find due to the similar spectral properties among them. In turn, C3 was identified as a Chl a isomer, belonging to the family of this compound, but without any other characteristic structural features. Finally, C6 was identified as pheophorbide a, since the loss of phytol group from the basic Chl a structure contributes to the shift of the characteristic peaks at 608 and 666 nm, with a secondary peak at 410 nm [25], as reported in Table 1 and presented in Figure 1.

In addition to chlorophylls, carotenoids are also found in the extracts of brown algae to a greater extent, being represented by xanthophylls and, secondarily, by carotenes. This prevalence of xanthophylls is a characteristic feature of brown algae, which are responsible not only for their coloration but also for most of their biological activities, together with polysaccharides [15,16,17,22]. A total of seven carotenoids, six xanthophylls (compounds X1–X6) and one carotene (compound B1) were reported in brown algae extracts (Table 1). Compound X1 was identified as fucoxanthin, according to the comparison to the analytical standard employed, thanks to its maximum peak at 450 nm and a negligible signal at 658 nm [26]. Compound X2 was identified as violaxanthin, as previously reported by other authors according to their absorption peaks at 418, 440 and 470 nm [27]. Auroxanthin was assigned to compound X3 based on its spectroscopic properties, which were previously determined by Dumont et al., showing a distinctive three-peak spectrum in the visible range at 380, 400 and 425 nm [28]. Moreover, compound X4 presents a prevalent peak at 442 nm together with a slight signal at 658, which is assumed to be a fucoxanthin derivative, as already reported in the extract of the microalgae *Tisochrysis lutea* [29]. Compound X5 exhibited a characteristic peak at 456 nm, compatible with the spectrum of dihydro lutein, which is already detected in extracts from algae belonging to the Prasinophyceae family [30]. Finally, compound X6 was identified as zeaxanthin, as previously described, based on the recorded maximum absorption peaks at 453 and 478 nm (Table 1) [28]. At last, the only carotene identified in algal extracts was β-carotene, according to its spectral characteristics, presenting two major peaks at 450 and 476 nm [31] and compared with the corresponding analytical standard.

**Figure 2:**
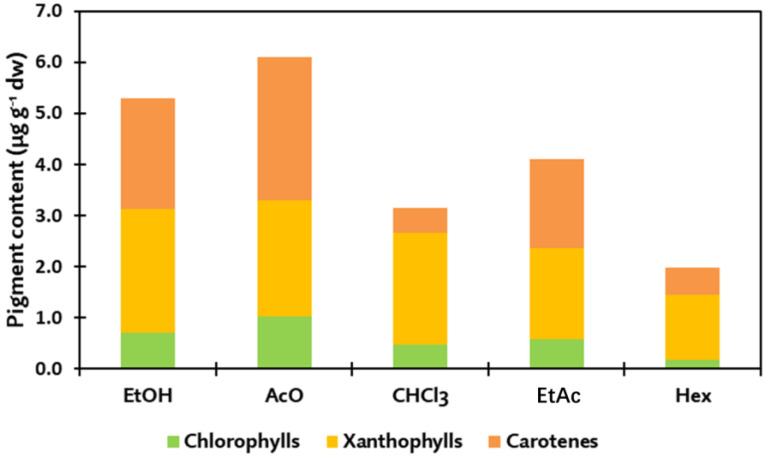
Content of pigment families extracted from the different by each solvent across the different brown algae analyzed. AcO, acetone; CHCl_3_, chloroform; EtAc, ethyl acetate; EtOH, ethanol; Hex, hexane.

Once identified, pigments were quantified individually and grouped into three different families: chlorophylls, xanthophylls and carotenes (Table 1). With the aim of comparing our results with those by other authors, Table 2 includes the pigments previously extracted from these algae, using different extraction solvents and determination methodologies. In general, organic solvents, such as acetone, methanol and ethanol, have been usually employed for the extraction of pigments from brown algae, whereas HPLC-DAD constitutes the most extended methodology involved in their determination, replacing the classical approach based on the spectrophotometric determination of pigments (Table 2). In the case of chlorophylls, C1 and C2 (chlorophylls c) were most abundant in the ethanolic extracts of UP, ranging 350–500 µg·g^−1^ dw, as well as in the ethanolic extracts of HE, whereas the Chl a family (compounds C3–C6) was found to exhibit the highest concentrations on LO and HE acetonic extracts, ranging 1.3–1.5 mg·g^−1^ dw (Table 1). Nevertheless, the greatest content of total chlorophylls was obtained in the ethanolic extracts of HE with ~2 mg·g^−1^ dw, followed by the acetonic extracts of HE, LO and SM, with chlorophylls contents of ~1.5 mg·g^−1^ dw in these cases. In absolute terms, chlorophylls were the family of pigments with the lowest contribution in brown algae, obtaining the best results using the most polar solvents used in this experimental procedure: ethanol and acetone (Figure 2). These results are in accordance with the previous literature that concluded acetone was the most efficient solvent to isolate chlorophylls from HE, UP and LO [32]. Other authors obtained similar results on chlorophyll c content from the aqueous acetonic extracts from UP, being effectively incorporated into sensitized solar cells [33]. Indeed, Chl c_2_ was described to be present in higher concentrations than Chl c_1_, which is in accordance with our data [34]. The better results for Chl c content on the ethanolic extracts of HE (500 µg·g^−1^ dw) with respect to acetone are also in line with previous results, suggesting that acetone is significantly less efficient than ethanol to extract chlorophyll c, as lower concentrations were reported by other authors, <400 µg·g^−1^ dw [35]. On the contrary, the results found for the acetonic extracts of SM showing a ~60% lower Chl c concentration compared with the acetonic SM extracts were already supported spectrophotometrically by Lewey and Gorham [36]. In terms of fresh algae, Chl c content ranged from 0.01 to 0.21 mg·g^−1^ of fresh weight (fw) for LO and AN, respectively, which reflect a lower content than Chl a, thus agreeing with the results reported for the nine algae analyzed. In the case of the Chl a family, other authors reported similar results for the acetonic extracts of HE, with only a 16% variation [35] compared with our results, whereas others reported up to 2.8 mg·g^−1^ dw using 90% aqueous acetone [37] and 2.7 mg·g^−1^ dw using pure acetone [36]. Differential results were also reported for LO and HE, for which their acetonic extracts showed the highest concentration in Chl a (~1.4 mg·g^−1^ dw), whereas a negligible 0.02 mg·g^−1^ dw was reported by Fernandes and co-workers in LO [35]. Regarding the equivalence in fresh weight, Chl a exhibited a concentration of <0.36 mg·g^−1^ fw for LO [38]. Such results indicate highly variable content in Chl a in brown algae, although there is wide evidence reflecting the efficient performance of 90% acetone as extracting solvent for chlorophylls (Table 2).

Xanthophylls were the most prevalent pigments in species AN, FS, LS, PC and UP (Table 1), fucoxanthin being the compound with the highest concentration, specifically in the ethanolic extracts of LS and the chloroform extracts of UP ranging ~3.9 mg·g^−1^ dw. There is wide evidence reporting that fucoxanthin is the most abundant pigment in brown algae [16], which supports the experimental results obtained. Indeed, fucoxanthin is the only pigment present in all extracts from all species, agreeing with the previous literature that admits this pigment as characteristic of brown algae [19]. The rest of the xanthophylls exhibited very low concentrations, accounting for >80% lower concentrations than fucoxanthin (Table 1). As a result, the highest yields in terms of xanthophylls were found on the same fucoxanthin-enriched extracts: the ethanolic and chloroform extracts of LS (4.7 and 4.3 mg·g^−1^ dw, respectively), the chloroform and acetone UP extracts (4.5 and 3.9 mg·g^−1^ dw, respectively). Due to the lower innocuity attributed to ethanol and acetone compared with chloroform, it is noteworthy that the employment of ethanolic extracts of LS or acetonic extracts of UP as biological producers of pigments is more efficient from a sustainable and nutritional point of view. They avoid the use of toxic and pollutant solvents for the extraction of these valuable compounds. Regarding xanthophyll yield, other authors agree with our results, proving that chloroform was the most efficient solvent to extract carotenoids from brown algae as HE and LO, for instance, with respect to other low-polarity solvents, such as hexane and diethyl ether [39]. Similar results have been found for fucoxanthin from UP using 75% aqueous ethanol as solvent (3.4 mg·g^−1^ dw) [40]. The isolation and purification of fucoxanthin also play significant roles in its yield, as purification via thin layer chromatography allowed reaching an impressive 18.6 mg·g^−1^ dw from the n-hexane:diethyl ether: chloroform (1:1:1, *v*:*v*:*v*) extract of HE [41]. Concerning the rest of xanthophylls, a lower concentration was reported in algal extracts analyzed in other works. Violaxanthin was the main secondary compound with rates from 4 µg·g^−1^ dw in methanolic extracts from LS [42] to 300 µg·g^−1^ dw in equivalent acetonic extracts [43]. Literature demonstrates that very different kinds of solvents have been employed for fucoxanthin extraction, ranging from water [44] to hexane, together with complex mixtures [45,46], which suggests that optimized protocols should be designed in order to effectively isolate this pigment from biological samples. Such observation is also reinforced by the results for fucoxanthin from fresh samples where a wide range of concentrations can be found depending on the alga species selected and the solvent used for extraction. In fact, the range of values proceeds from 0.05 mg·g^−1^ fw for the acetonic extracts of HE [47] to 0.39 mg·g^−1^ fw for the dimethyl ether extracts of UP, which was reported as the species with the highest concentration [48].

As stated before, carotenes were exclusively represented by β-carotene, and it was the most prevalent pigment in BB, HE, LO and SM extracts (Table 1), whereas it was absent in acetonic and ethyl acetate extracts from FS, which contrasts with previous findings [54]. Indeed, BB extracts performed with ethyl acetate were the ones reflecting the highest concentration of 7.2 mg·g^−1^ dw, followed by the acetonic LO extracts and ethanolic HE extracts obtaining comparable results, ~6.2 µg−g^−1^ dw. On the contrary, an absence of carotene was observed in PC extracts, and negligible concentrations were reported in the case AN and FS extracts. Noteworthy, our results displayed that the highest concentrations of this compound were obtained with different solvents, such as ethyl acetate, ethanol and acetone (Table 1). Such a difference in β-carotene solubility depending on the extraction solvent has been suggested to be a function of its isomerization degree, presenting different proportions of Z-isomers and E-isomers, as proven for acetone, hexane and ethanol [68]. Results from other authors are rather different in terms of β-carotene content, showing maximum values of 0.24 mg·g^−1^ dw in the hexane extracts of PC [59] and 0.32 mg·g^−1^ dw for the acetonic extracts of LS [44]. Concerning fresh materials, microalgae exhibit high carotene contents, reaching values up to 1.2 mg·g^−1^ fw, being 20-times higher than in brown seaweeds, which ranged 2–54 µg·g^−1^ fw [32]. Accordingly, as recently indicated for red seaweeds, the presence of β-carotene is thought to be highly dependent on the light intensity alga receives, as it plays a major role in the photoprotection of these organisms [69], which may be responsible for the great differences reported here.

For the sake of simplicity, an integrated representation of pigment contents depending on the extraction solvent is shown in Figure 2. In general terms, chlorophylls constitute the pigment family with the lowest concentration for all brown algae, followed by the carotenoid family, for which xanthophylls and carotenes show a differential presence in algal extracts. In total, the highest contents of pigments were obtained for the ethanolic extracts of HE, with 11.9 mg·g^−1^ dw and acetonic extracts of LO (10.3 mg·g^−1^ dw), whereas PC extracts showed the lowest results, with maximal values located below 2 mg·g^−1^ dw for all solvents (Table 1). The abundance and proportion of different pigments are results of biological factors, showing a species-dependent composition. Furthermore, there are additional causes, including geographical and season-related factors, together with age, depth, nutrient availability, salinity or UV light exposure that play a significant role on algal pigment production, as part of the defensive and adaptative responses of these organisms against both abiotic and biotic stresses [70]. It is important to note that these reported pigments are not unique in brown algae, since there are other pigmented compounds present in these species, represented by the water soluble phycobiliproteins, divided mainly into phycocyanins and phycoerythrins [32]. Moreover, although they are not considered as pigments, phlorotannins also promote valuable protection against intense UV radiation, coordinating such roles with that of pigments as characteristic phenolic compounds from brown algae [71,72]. Accordingly, the presence of these compounds should be also taken into account when referring to the total pigment content of macroalgae. On these bases, the combination of environmental, physicochemical and biological factors drives the accumulation of pigments in brown algae following a multifactorial trend, as suggested by our results.

Concerning solvents, ethanol and acetone were the most efficient for chlorophyll and carotene extraction, while chloroform is likely to be the best one for recovering xanthophylls (Figure 2). Such efficiency is mostly driven by the solubility of target compounds on the organic solvents employed in their extraction. Thus, it was expected that ethanol and acetone provide a similar extraction efficiency, since they present almost the same polarity index units, 5.2 and 5.1, respectively, although they present a number of different structural features, such as viscosity and atomic configuration since acetone is considered a dipolar aprotic solvent whereas ethanol is a polar protic solvent [73]. Indeed, a significant correlation (*p* < 0.05) between solvent polarity and pigment content was reported, reflecting a statistical assessment of the efficiency of polar solvents as extractants of brown algae pigments. This behavior was widely assessed by many authors in other macroalgal species motivated by two major causes: i) Pigments show an enhanced affinity towards polar organic solvents because of their solubility, guided by their structural features, which include several polar modifications; and ii) such polar solvents have been reported as efficient disruptors of algal cells through the dissolution of cell wall membranes, facilitating the release of intracellular components [74]. In the case of xanthophylls, comparable results were obtained using chloroform or ethanol depending on the species (Figure 2). This could be due to the prevalence of fucoxanthin within this family of compounds, as it is assumed to be extracted and purified using less polar solvents, including chloroform, as it was observed for HE by Rajauria et al. [41].

Regarding the heterogeneity found for the extraction of pigments among distinct species, in addition to biological factors, the extraction methodology also plays a paramount role. The heat-assisted solid–liquid extraction employed in this study has been classically applied for the extraction of pigments from macroalgae [74]. Therefore, the transference of pigments from extracted samples was exclusively guided by the polarity of solvents, helped by an increase in temperature, as stated earlier. Novel extraction techniques are replacing such classical approaches in the last years, including a wide range of methodologies, such as enzyme, microwave, ultrasound, pressurized liquid and supercritical fluid assisted extractions. Most of them have already been applied to brown algae and are characterized for forcing the dissolution of compounds into the solvents used for extraction [13]. These techniques not only help the extraction of pigments without interfering with their stability but are also faster and compatible with the use of safe solvents; thus, they are known as green techniques. The performance of these extractive methodologies, in consequence, is resulting in the adoption of more sustainable workflows for the production of bioactive compounds from biological sources, and their application to algal pigments is claiming the interest of economically important industries in this century, as it is the case of biorefineries [75]. Moreover, the application of green extraction techniques supplies an added advantage, since they show excellent plasticity, being easily transferred from the laboratory to the industrial scale, thus urging the production of algal pigments. Among them, the use of natural deep eutectic solvents for the isolation of algal pigments is gaining popularity in the last years for the simultaneous extraction of either hydrophilic and hydrophobic compounds with high yields [76].

## 3. Materials and Methods

### 3.1. Sample Collection

The species involved in this study, which include all brown algae belonging to Phaeophyceae family, were recollected manually from the Galician coastline (NW Spain) in winter 2019 and provided by Algamar (www.algamar.com; accessed on 13 December 2021): *Ascophyllum nodosum* (L.) Le Jolis, AN; *Bifurcaria bifurcata* R. Ross, BB; *Fucus spiralis* (L.), FS; *Himanthalia elongata* (L.) S.F.Gray, HE; *Laminaria ochroleuca* de la Pylaie, LO; *Laminaria saccharina* (L.) Lamouroux, LS; *Pelvetia canaliculata* (L.) Decne. Thur., PC; *Sargassum muricum* (Yendo) Fensholt, SM; and *Undaria pinnatifida* (Harvey) Suringar, UP (Table 3).

After collection, samples were washed with abundant tap water, stored in plastic zip bags at −80 °C and further lyophilized (LyoAlfa 10/15 from Telstar), crushed and sieved. The obtained powdered material was stored at −20 °C until use.

### 3.2. Sample Extraction

Dried algae (0.6 g) were mixed with 20 mL of the corresponding solvent: ethanol (EtOH), acetone (AcO), chloroform (CHCl_3_), ethyl acetate (EtAc) and hexane (Hex). The mixture was incubated at 50 °C in an orbital shaker at 150 rpm for 24 h in the dark. The supernatant was removed, and the pellet was re-extracted twice with 10 mL of solvent using the same conditions described before but for one hour; thus, the final solid:liquid ratio achieved was 30 g/L. Afterwards, the total volume was collected and centrifuged at 4800 rpm for 8 min to eliminate the remaining solid particles. To find the extraction yield, 5 mL of such extracts was transferred to crucibles and incubated for 24 h at 104 °C until complete dryness. The dry residue was then weighted, and the extraction yield was expressed as the percentage of dry residue with respect to dry original material.

In parallel, the remaining extract was later concentrated in a rotary evaporator at 40 °C to obtain the dry extracts. Dry extracts were then resuspended in 10 mL of 80% aqueous ethanol, syringe filtered (0.22 µm pore size) into amber vials and stored at −20 °C until their further analysis.

### 3.3. Chromatographic Analysis of Algal Pigments via HPLC-DAD

The identification and quantification of the pigments in the prepared extracts was performed by using high performance liquid chromatography coupled to a diode array detector (HPLC-DAD) using the Agilent 1260 Infinity HPLC equipment (Santa Clara, CA, USA) (including a 2690 separations module (low-pressure mixing system) and a Waters 996 diode-array detector (Milford, MA, USA; 1.2 nm optical resolution) interfaced with a Waters 474 scanning fluorescence detector by means of a Sat/In analog interface). The column used for chromatographic separation was a Waters Symmetry C8 column (150 mm × 4.6 mm, 3.5 pm particle size and 100 Å pore size), which was thermostatted at 25 °C by using a refrigerated circulator water bath (Neslab RTE-200; Milano, Italy) connected to an HPLC column water jacket (Alltech). The mobile phase consisted of two different eluents: a mixture of methanol:acetonitrile:0.25 M aqueous pyridine (50:25:25, *v*:*v*:*v*) as eluent A; and a mixture methanol:acetonitrile:acetone (20:60:20, *v*:*v*:*v*) as eluent B. All solvents used were HPLC grade. Flow rate was set at 1 mL/min for the entire run following the following elution gradient: 0–40% B (22 min), 40–95% B (6 min) and isocratic 95% B (12 min).

Pigments were spectrophotometrically detected by DAD at a wavelength of 440 nm. The identification was conducted by comparing the UV-Vis spectra of each peak individually recorded with the previous literature. For quantification, three different standards were used to obtain the corresponding calibration curves: chlorophyll a, fucoxanthin and β-carotene, all purchased from Sigma with a purity ≥95% (HPLC grade). This HPLC-DAD protocol was conducted in SSADS-CACTI (Food Security and Sustainable Development Laboratory, Scientific and Technological Support Centre for Research—University of Vigo, Spain).

The identified compounds were then grouped into three general families according to their biosynthetic origin: chlorophylls, xanthophylls and carotenes. Thus, the chlorophylls family included chlorophyll a, chlorophyll a epimer, chlorophyll a allomer, chlorophyll c1, chlorophyll c2 and pheophorbide A, which were quantified according to chlorophyll a calibration curve (y = 103.742x − 71.610; *R^2^* = 0.999; LOD = 0.10 µg/mL; LOQ = 0.34 µg/mL). The xanthophylls family included fucoxanthin, violaxanthin, auroxanthin, fucoxanthin derivative, dihydrolutein and zeaxanthin, and they were quantified according to fucoxanthin calibration curve (y = 84.986x + 15.508; *R^2^* = 1.000; LOD = 0.16 µg/mL; LOQ = 0.54 µg/mL). Finally, the carotene family was only represented by β-carotene, which was quantified according to its calibration curve (y = 1.131x − 1.372; *R^2^* = 0.998; LOD = 1.22 µg/mL; LOQ = 4.08 µg/mL). The results were expressed in microgram (µg) per gram of alga dry weight (µg·g^−1^ dw).

### 3.4. Statistical Analysis

The relative standard deviation (RSD) between chromatographic analyses was assessed for each of the three standards used, and it was <5% in all cases. The correlation between solvent polarity index and pigment content was statistically analyzed by Pearson’s correlation coefficient. The significance level was adjusted at *α* = 0.05.

## 4. Conclusions

In this study, the extraction and HPLC-DAD identification of pigments from nine brown algae using five different extraction solvents showed that these marine organisms present a heterogeneous composition, including xanthophylls, especially xanthophylls such as fucoxanthin, and carotenes, such as β-carotene, as the most prevalent pigments, followed by chlorophylls at a lesser extent. Fucoxanthin was present in all the extracts obtained, being effectively extracted using organic solvents permitted for human consumption, such as ethanol or acetone. In global terms, the algae *L. saccharina* and *U. pinnatifida* showed the highest fucoxanthin contents. Overall, ethanol and acetone were revealed as the most efficient solvents for pigment extraction, being also effective on the extraction of chlorophylls a and c and β-carotene. Regarding the sum of all pigments, the ethanolic extracts of *H. elongata* and the acetonic extracts of *L. ochroleuca* exhibited the highest results, accounting for more than 11 and 10 mg·g^−1^ dw, respectively. In the same manner, ethanol supplied the highest rates of extraction yield for all species analyzed, being reported as the most efficient solvent to perform a first screening of chemical composition of algae. Therefore, it was proved that brown algae are not only a promising source of pigments but also bioactive compounds, since fucoxanthin has been assessed as a potent health-enhancing compound thanks to their associated antioxidant, antimicrobial, anticancer and neuroprotective properties, among others. All these features attributed to brown algae pigments open a wide perspective on several industries including those related to food, cosmetic and pharmaceutical sectors, where a plethora of applications has been proposed: ranging from the coloration of matrices and products to the fortification of foods resulting in their functionalization and their incorporation to cosmetical preparations due to their properties as UV protectants and anti-aging molecules.

## Figures and Tables

**Table 1:** Extraction yield (%), pigment content (µg g^−1^ dw) ^1^ of the nine brown algae according to different solvents used for extraction, and HPLC parameters involved in their identification.

Algae	Solvent	Yield (%)	Chlorophylls							Xanthophylls							Carot.	Total
			C1	C2	C3	C4	C5	C6	Total	X1	X2	X3	X4	X5	X6	Total	B1	
AN	*EtOH*	18.15	20.6	-	34.4	31.6	35.6	23.1	145.3	623.9	-	-	132.1	-	2.6	758.7	-	904.0
	*AcO*	15.38	64.2	17.6	170.8	343.8	21.8	30.1	648.2	940.9	-	8.7	201.0	8.6	-	1159.1	1085.4	2892.7
	*CHCl_3_*	9.43	20.8	20.8	90.7	127.2	-	16.1	275.7	696.8	1.3	6.3	147.5	9.4	-	861.3	-	1137.0
	*EtAc*	10.31	-	-	61.5	106.5	19.7	20.4	208.1	697.2	-	6.8	147.6	0.4	-	852.0	-	1060.1
	*Hex*	7.99	-	-	32.7	42.9	-	-	75.6	673.4	129.9	21.9	40.0	17.0	-	882.2	-	957.8
BB	*EtOH*	24.15	32.9	21.7	-	-	-	36.9	91.6	806.2	-	-	146.0	-	132.7	1084.9	3168.7	4345.2
	*AcO*	10.85	37.8	28.7	-	357.1	68.0	36.0	527.6	623.0	-	-	187.2	-	107.7	917.8	4758.4	6203.8
	*CHCl_3_*	4.43	-	-	-	210.8	35.7	27.9	274.3	631.9	-	-	164.6	-	100.6	897.0	1354.1	2525.4
	*EtAc*	4.43	28.4	22.3	-	323.4	45.9	54.4	474.4	736.3	-	-	186.5	-	130.6	1053.4	7261.5	8789.3
	*Hex*	1.57	-	-	-	167.3	50.4	-	217.7	271.5	-	-	50.5	-	79.0	401.0	-	618.7
FS	*EtOH*	14.63	19.3	-	-	-	-	39.7	58.9	1171.3	-	-	233.9	-	5.2	1410.3	-	1469.2
	*AcO*	7.75	79.7	49.6	212.0	471.5	23.3	-	836.1	1417.4	-	10.5	278.9	45.3	19.3	1771.5	-	2607.6
	*CHCl_3_*	7.03	-	-	-	135.6	191.9	20.5	348.0	1547.7	13.3	23.3	276.4	26.1	-	1886.8	-	2234.8
	*EtAc*	5.31	55.7	22.6	-	97.6	162.4	19.0	357.2	1490.9	33.2	30.1	267.8	6.7	-	1828.8	-	2186.0
	*Hex*	5.75	-	-	-	45.8	66.1	22.9	134.8	1008.7	215.7	44.8	120.8	-	1.8	1391.7	446.3	1972.8
HE	*EtOH*	26.99	500.1	176.3	35.6	1039.6	126.0	50.7	1928.3	3114.1	-	37.6	543.1	52.0	119.5	3866.4	6141.7	11,936.4
	*AcO*	3.60	140.6	65.9	20.9	1308.2	105.4	32.1	1673.0	2985.5	163.8	60.1	512.9	38.2	116.4	3876.8	4328.7	9878.6
	*CHCl_3_*	3.15	-	-	-	719.4	52.4	24.8	796.5	2771.3	109.4	30.3	517.3	44.9	106.0	3579.2	1670.3	6046.0
	*EtAc*	0.15	52.4	31.9	-	819.5	61.6	25.3	990.8	1765.5	57.3	55.3	346.9	-	65.2	2290.2	3186.3	6467.2
	*Hex*	2.10	-	-	-	352.1	111.5	33.0	496.6	1512.0	235.9	48.1	178.0	2.7	57.9	2034.6	2782.4	5313.6
LO	*EtOH*	19.2	16.6	18.0	172.4	432.8	570.4	74.2	1284.4	2926.9	-	-	237.9	2.8	-	3167.6	4571.9	9023.9
	*AcO*	0.8	-	-	25.7	1393.9	83.5	-	1503.1	2427.7	143.7	18.6	498.6	10.1	-	3098.7	5675.5	10277.3
	*CHCl_3_*	2.1	-	-	55.7	398.2	28.0	16.9	498.8	697.3	51.2	-	236.5	-	-	985.0	470.2	1954.0
	*EtAc*	1.4	-	-	19.3	968.0	64.1	23.0	1074.4	1709.0	83.6	7.2	549.1	-	-	2349.0	1044.6	4468.0
	*Hex*	0.6	-	-	26.7	73.3	17.6	-	117.6	871.9	65.1	-	325.2	-	-	1262.2	-	1379.7
LS	*EtOH*	17.97	35.5	69.8	628.8	604.5	131.8	64.9	1535.3	3904.8	-	69.9	687.5	5.6	-	4667.9	2895.7	9098.8
	*AcO*	2.92	25.1	38.3	36.6	976.3	128.3	25.4	1229.9	1844.4	102.0	29.9	388.8	-	-	2365.1	2587.8	6182.8
	*CHCl_3_*	1.69	-	-	72.9	585.0	123.6	36.3	817.9	3344.7	72.3	55.4	769.2	-	27.5	4269.0	-	5086.9
	*EtAc*	0.61	-	-	39.0	352.1	48.9	23.4	463.4	1264.0	10.7	17.4	416.3	-	5.0	1713.4	1202.3	3379.1
	*Hex*	1.38	-	-	30.8	51.6	28.2	20.8	131.4	670.7	58.4	3.7	256.9	-	-	989.6	-	1121.0
PC	*EtOH*	15.63	53.6	33.0	-	199.3	28.8	22.2	336.9	717.6	-	11.1	137.2	-	0.4	866.3	-	1203.2
	*AcO*	12.18	70.4	42.9	16.5	247.4	19.7	18.5	415.3	923.1	11.1	17.3	192.3	4.0	16.0	1163.9	-	1579.2
	*CHCl_3_*	7.59	-	-	-	81.2	108.4	20.9	210.5	1487.1	-	0.1	241.1	-	-	1728.4	-	1938.9
	*EtAc*	7.74	22.6	19.7	-	226.6	16.6	17.9	303.3	643.6	7.5	11.1	137.1	1.8	10.7	811.8	-	1115.2
	*Hex*	6.87	-	-	-	115.8	27.3	17.2	160.4	815.1	309.7	35.0	119.2	-	15.5	1294.5	-	1454.8
SM	*EtOH*	17.9	58.1	21.1	-	-	-	37.1	116.3	2322.5	-	-	437.1	-	9.3	2768.8	-	2885.1
	*AcO*	3.4	184.4	116.1	313.6	828.0	51.1	62.0	1555.2	1735.4	23.2	9.5	381.9	32.2	18.2	2200.4	3930.9	7686.5
	*CHCl_3_*	2.1	-	-	26.3	354.0	30.8	15.8	426.8	826.9	16.5	-	261.7	-	-	1105.1	851.7	2383.7
	*EtAc*	0.3	15.7	19.4	36.3	174.8	315.2	-	561.5	1591.8	6.8	2.8	359.7	1.5	-	1962.6	-	2524.1
	*Hex*	2.0	-	-	80.8	142.4	41.3	-	264.5	897.1	79.2	5.7	132.0	-	-	1113.9	-	1378.5
UP	*EtOH*	38.84	504.3	346.7	-	33.7	-	26.7	911.3	2576.3	-	16.3	432.5	2.1	54.8	3082.0	2839.8	6833.2
	*AcO*	3.35	-	35.2	-	773.0	49.0	26.7	883.9	3310.7	-	45.0	597.2	-	-	3952.8	2792.4	7629.1
	*CHCl_3_*	3.22	55.8	56.6	174.3	249.0	-	24.7	560.4	3777.8	-	3.8	707.3	4.7	21.8	4515.4	-	5075.8
	*EtAc*	2.38	35.6	39.6	-	624.1	34.5	36.0	769.9	2536.2	-	26.7	545.5	-	35.1	3143.5	3147.7	7061.1
	*Hex*	2.24	-	-	-	38.6	-	30.2	68.7	1705.3	-	28.5	212.2	29.9	28.6	2004.5	1639.3	3712.5
HPLC parameters		*RT (min)*	9.88	10.5	32.1	32.4	32.7	34.2	-	16.8	19.5	21.4	22.5	24.2	26.8	-	34.6	-
		*λ max (nm)*	452, 584, 634	448, 580, 632	430, 616, 662	430, 616, 664	430, 618, 664	410, 608, 666	-	450, 658	418, 440, 470	382, 402, 428	442, 658	402, 426, 456, 658	(424), 452, 478	-	(416), 450, 476	-

Abbreviations: Carot., carotenes; C1, chlorophyll c2; C2, chlorophyll c1; C3, chlorophyll a isomer; C4, chlorophyll a; C5, chlorophyll a isomer; C6, pheophorbide A; X1, fucoxanthin; X2, violaxanthin; X3, auroxanthin; X4, fucoxanthin derivative; X5, Dihydrolutein; X6, Zeaxanthin; B1, β-carotene; RT, retention time; λ max, maximum wavelength. ^1^ The quantification of all chlorophylls (compounds C1–C6) was determined according with the calibration curve of Chl a; the quantification of all xanthopylls (compounds X1–X6 was determined according with the calibration curve of xanthophyll; the quantification of carotenes (compound C1) was determined according with the calibration curve of β-carotene.

**Table 2:** Pigment contents (mg per g of dry alga) ^1^ and extraction conditions of the nine brown algae involved in this study.

Species ^2^	Extraction Solvent ^3^ // Determination ^4^	Compounds ^5^					Ref.
		Chl c	Chl a	Fx	Vx	Bcar	
AN	Several // HPLC-DAD		1.0	0.66	0.13	0.1	[49]
	Several // HPLC-DAD			1.78			[50]
	AcO:water (9:1, *v*:*v*) // HPLC-DAD		1.34				[6]
	AcO:water (9:1, *v*:*v*) // HPLC-DAD		3.66	1.8		0.5	[37]
	AcO:water (9:1, *v*:*v*) // HPLC-DAD	0.3	1.5	0.7		1.1	[35]
	AcO // HPLC-DAD			0.4			[51]
FS	MeOH // UV-Vis		3.0				[52]
	MeOH:Hex (1:4, *v*:*v*) // UV-Vis		0.0005 *	0.171 *		0.007 *	[53]
	MeOH // UV-Vis			0.171 *			[54]
HE	MeOH // UV-Vis		0.63				[32]
	MeOH// UV-Vis		0.60				[32]
	EtOH // UV-Vis		0.68				[32]
	AcO // UV-Vis		1.57				[32]
	DMSO:water (4:1, *v*:*v*) // UV-Vis			0.28			[32]
	Hex:EtOEt:CHCl_3_ (1:1:1, *v*:*v*:*v*) // LC-DAD-ESI-MS and NMR			18.60			[41]
	AcO:water (9:1, *v*:*v*) // HPLC-DAD		2.8	3.3		0.58	[37]
	AcO // HPLC–DAD			0.05 *			[47]
	AcO:water (9:1, *v*:*v*) // HPLC-DAD	0.4	1.6	1.1		1.5	[35]
	MeOH:Hex:CH_2_Cl_2_ (50:25:25, *v*:*v*:*v*) // HPLC-MS		1.5	0.009		0.004	[45]
	MeOH:Hex: CH_2_Cl_2_ (50:25:25, *v*:*v*:*v*) // HPLC-DAD		0.043	0.051		0.0095	[46]
	AcO // HPLC-DAD			0.3			[51]
LO	DMF // HPLC-DAD	0.08 *	0.36 *			0.01 *	[38]
	AcO // HPLC-DAD		0.018	0.163	0.006	0.005	[37]
LS	MeOH // HPLC-DAD		0.183				[55]
	MeOH // HPLC-DAD		0.655	0.665	0.036	0.023	[42]
	MeOH // UV-Vis		0.143				[32]
	MeOH// UV-Vis		0.111				[32]
	EtOH // UV-Vis		0.114				[32]
	AcO:water (9:1, *v*:*v*) // HPLC-DAD		1.35	0.59	0.02	0.03	[56]
	AcO // HPLC–DAD			0.016			[47]
	AcO:water (9:1, *v*:*v*) // HPLC-DAD		0.09	0.08			[57]
	AcO // UV-Vis		0.184				[32]
	AcO // HPLC-DAD		0.029	0.433	0.302	0.316	[43]
	AcO:water (7:3, *v*:*v*) // UV-Vis	0.38	0.58				[58]
PC	Hex // UV-Vis		0.602			0.236	[59]
	AcO // UV-Vis		1.2 *				[60]
SM	AcO // UV-Vis	0.440	2.720			0.080	[36]
	AcO // UV-Vis		0.4				[61]
	AcO:water (8:2, *v*:*v*) // UV-Vis		2.1 *				[62]
UP	MeOH // UV-Vis		0.349				[32]
	MeOH // UV-Vis		0.331				[32]
	MeOH // HPLC-DAD					0.013	[63]
	MeOH // HPLC-DAD			0.728			[64]
	MeOH // HPLC-DAD			5.0			[65]
	EtOH // UV-Vis		0.321				[32]
	EtOH:water (8:2, *v*:*v*) // UV-Vis					0.008	[66]
	EtOH:water (3:1, *v*:*v*) // HPLC-DAD			3.37			[40]
	AcO // UV-Vis		0.543				[32]
	AcO // HPLC-DAD			2.3			[67]
	DMF // UV-Vis		0.437				[32]
	DMSO:water (4:1, *v*:*v*) // UV-Vis			0.014			[32]
	Water // HPLC-DAD			0.73			[44]
	EtOEt // HPLC-DAD			0.39 *			[48]

^1^ Concentrations are expressed in mg g^−1^ dry alga, unless otherwise stated: * mg g^−1^ fresh alga. ^2^ Species: AN, *Ascophyllum nodosum*; FS, *Fucus spiralis*; HE, *Himanthalia elongata*; LO, *Laminaria ochroleuca*; LS, *Laminaria saccharina*; PC, *Pelvetia canaliculata*; SM, *Sargassum muticum*; UP, *Undaria pinnatifida*. ^3^ Solvents: AcO, acetone; CHCl3, chloroform; CH2Cl2, dichloromethane; DMF, dimethyl formamide; DMSO, dimethyl sulfoxide; EtOEt, diethyl ether; EtOH, ethanol; Hex, hexane; MeOH, methanol. ^4^ Determination methodologies: DAD, diode array detector; ESI, electrospray ionization; GC, gas chromatography; HPLC, high performance liquid chromatography; LC, liquid chromatography; MS, mass spectrometry; NMR, nuclear magnetic resonance; UV-Vis, ultraviolet-visible spectrometry. ^5^ Compounds: Bcar, β-carotene; Chl a, chlorophyll a; Chl c, chlorophyll c; Fx, fucoxanthin; Vx, violaxanthin.

**Table 3:** Relevant information of the nine brown algae species from Phaeophyceae phylum investigated in this study [77,78].

Species	Family	Common Name(s)	Distribution	Depth	Habitat Preferences
*Ascophyllum nudosum*	Fucaceae	Rockweed, Norwegian kelp, Egg wrack	North Atlantic Ocean	Not relevant	Sheltered rocky shores, intertidal habit.
*Bifurcaria bifurcata*	Fucaceae	Brown Tuning Fork Weed, Brown Forking Weed	From Ireland to Senegal.	Ponds	Rock pools on the middle and lower shore, particularly on exposed beaches. It also forms a low water zone in some locations in Southwest England and West Ireland.
*Fucus spiralis*	Fucaceae	Spiral wrack, Flat wrack	North Atlantic Ocean and isolated reports in the Northern Pacific.	Not relevant	Rocky substrata on sheltered to moderately exposed shores, intertidal habit.
*Himanthalia elongata*	Fucaceae	Sea spaghetti	From Norway to Portugal	Intertidal and infralittoral zone	Forming a very characteristic band
*Laminaria saccharina*	Laminariaceae	Sugar kelp, Sea belt, Devil’s apron	Arctic Ocean down to Northern Portugal	Sublittoral zone (max 30 m)	Moderately to sheltered sites, often on unstable substrate (boulders, mussels and rocks)
*Laminaria orcholeuca*	Laminariaceae	Kelp	Arctic and Atlantic Oceans	Lower littoral and sublittoral zone (max 20 m)	Exposed to moderately exposed sites, hard substrate and strong currents
*Pelvetia canaliculata*	Fucaceae	Channelled wrack, Cow tang	European coastline	Supralittoral zone	Sheltered to moderately exposed, hard substrate and high tolerance of desiccation
*Sargassum muticum*	Fucaceae	Japanese wireweed	Atlantic and Pacific Oceans	-	Hard substrata in shallow waters
*Undaria pinnatifida*	Laminariaceae	Wakame	Northern Europe, Argentina, Mexico, Australia, New Zealand, Japan, Korea and China	Infralittoral	Grows on stones, epiphyte or artificial structures (swamps and ship hulls)

## Data Availability

Not applicable.

## References

[1] Mesías F.J., Martín A., Hernández A. (2021). Consumers’ growing appetite for natural foods: Perceptions towards the use of natural preservatives in fresh fruit. Food Res. Int..

[2] Silva A., Rodrigues C., Garcia-Oliveira P., Lourenço-Lopes C., Silva S.A., Garcia-Perez P., Carvalho A.P., Domingues V.F., Barroso M.F., Delerue-Matos C. (2021). Screening of bioactive properties in brown algae from the northwest iberian peninsula. Foods.

[3] Losada-Lopez C., Dopico D.C., Faína-Medín J.A. (2021). Neophobia and seaweed consumption: Effects on consumer attitude and willingness to consume seaweed. Int. J. Gastron. Food Sci..

[4] De Carvalho J.C., Cardoso L.C., Ghiggi V., Woiciechowski A.L., De Souza Vandenberghe L.P., Soccol C.R. (2014). Microbial Pigments. Biotransformation of Waste Biomass into High Value Biochemicals.

[5] Kuda T., Nishizawa M., Toshima D., Matsushima K., Yoshida S., Takahashi H., Kimura B., Yamagishi T. (2021). Antioxidant and anti-norovirus properties of aqueous acetic acid macromolecular extracts of edible brown macroalgae. LWT.

[6] Schmid M., Guihéneuf F., Nitschke U., Stengel D.B. (2021). Acclimation potential and biochemical response of four temperate macroalgae to light and future seasonal temperature scenarios. Algal Res..

[7] Lourenço-Lopes C., Fraga-Corral M., Jimenez-Lopez C., Carpena M., Pereira A.G., Garcia-Oliveira P., Prieto M.A., Simal-Gandara J. (2021). Biological action mechanisms of fucoxanthin extracted from algae for application in food and cosmetic industries. Trends Food Sci. Technol..

[8] Pereira A.G., Otero P., Echave J., Carreira-Casais A., Chamorro F., Collazo N., Jaboui A., Lourenço-Lopes C., Simal-Gandara J., Prieto M.A. (2021). Xanthophylls from the Sea: Algae as Source of Bioactive Carotenoids. Mar. Drugs.

[9] Pereira A.G., Fraga-Corral M., Garcia-Oliveira P., Lourenço-Lopes C., Carpena M., Prieto M.A., Simal-Gandara J. (2021). The use of invasive algae species as a source of secondary metabolites and biological activities: Spain as case-study. Mar. Drugs.

[10] Park J.J., Lee W.Y. (2021). Anti-glycation effects of brown algae extracts and its phenolic compounds. Food Biosci..

[11] Yuan Y., Zhang J., Fan J., Clark J., Shen P., Li Y., Zhang C. (2018). Microwave assisted extraction of phenolic compounds from four economic brown macroalgae species and evaluation of their antioxidant activities and inhibitory effects on α-amylase, α-glucosidase, pancreatic lipase and tyrosinase. Food Res. Int..

[12] Kumar Y., Singhal S., Tarafdar A., Pharande A., Ganesan M., Badgujar P.C. (2020). Ultrasound assisted extraction of selected edible macroalgae: Effect on antioxidant activity and quantitative assessment of polyphenols by liquid chromatography with tandem mass spectrometry (LC-MS/MS). Algal Res..

[13] Lourenço-Lopes C., Garcia-Oliveira P., Carpena M., Fraga-Corral M., Jimenez-Lopez C., Pereira A.G., Prieto M.A., Simal-Gandara J. (2020). Scientific approaches on extraction, purification and stability for the commercialization of fucoxanthin recovered from brown algae. Foods.

[14] Aryee A.N., Agyei D., Akanbi T.O. (2018). Recovery and utilization of seaweed pigments in food processing. Curr. Opin. Food Sci..

[15] Li Y., Zheng Y., Zhang Y., Yang Y., Wang P., Imre B., Wong A.C.Y., Hsieh Y.S.Y., Wang D. (2021). Brown algae carbohydrates: Structures, pharmaceutical properties, and research challenges. Mar. Drugs.

[16] Li Y., Qin J., Cheng Y., Lv D., Li M., Qi Y., Lan J., Zhao Q., Li Z. (2021). Marine sulfated polysaccharides: Preventive and therapeutic effects on metabolic syndrome. Mar. Drugs.

[17] Pozharitskaya O.N., Obluchinskaya E.D., Shikov A.N. (2020). Mechanisms of bioactivities of fucoidan from the brown seaweed *Fucus vesiculosus* L. of the Barents Sea. Mar. Drugs.

[18] Food and Drug Administration. https://www.govinfo.gov/app/details/CFR-2012-title21-vol3/CFR-2012-title21-vol3-sec184-1120/summary.

[19] André R., Pacheco R., Bourbon M., Serralheiro M.L. (2021). Brown algae potential as a functional food against hypercholesterolemia: Review. Foods.

[20] Smith J.L., Summers G., Wong R. (2010). Nutrient and heavy metal content of edible seaweeds in New Zealand. N. Z. J. Crop Hortic. Sci..

[21] Tsubaki S., Oono K., Hiraoka M., Onda A., Mitani T. (2016). Microwave-assisted hydrothermal extraction of sulfated polysaccharides from *Ulva* spp. and *Monostroma latissimum*. Food Chem..

[22] Büchel C. (2020). Light harvesting complexes in chlorophyll c-containing algae. Biochim. Biophys. Acta Bioenerg..

[23] Singh A.K., Rana H.K., Pandey A.K. (2020). Analysis of Chlorophylls.

[24] Zeb A., Haq A., Murkovic M. (2019). Effects of microwave cooking on carotenoids, phenolic compounds and antioxidant activity of *Cichorium intybus* L. (chicory) leaves. Eur. Food Res. Technol..

[25] Činčárová D., Hájek J., Dobřichovský M., Lukeš M., Hrouzek P. (2021). Recommendations on the quantitative analysis of pheophorbides, photosensitizers present in algal biomass intended as food supplement. Algal Res..

[26] Erdoğan A., Karataş A.B., Demirel Z., Dalay M.C. (2021). Purification of fucoxanthin from the diatom *Amphora capitellata* by preparative chromatography after its enhanced productivity via oxidative stress. J. Appl. Phycol..

[27] Steingass C.B., Vollmer K., Lux P.E., Dell C., Carle R., Schweiggert R.M. (2020). HPLC-DAD-APCI-MSn analysis of the genuine carotenoid pattern of pineapple (*Ananas comosus* [L.] Merr.) infructescence. Food Res. Int..

[28] Dumont D., Danielato G., Chastellier A., Saint Oyant L.H., Fanciullino A.L., Lugan R. (2020). Multi-targeted metabolic profiling of carotenoids, phenolic compounds and primary metabolites in goji (*Lycium* spp.) berry and tomato (*Solanum lycopersicum*) reveals inter and intra genus biomarkers. Metabolites.

[29] Gallego R., Tardif C., Parreira C., Guerra T., Alves M.J., Ibáñez E., Herrero M. (2020). Simultaneous extraction and purification of fucoxanthin from *Tisochrysis lutea* microalgae using compressed fluids. J. Sep. Sci..

[30] Latasa M., Scharek R., Le Gall F., Guillou L. (2004). Pigment suites and taxonomic groups in Prasinophyceae. J. Phycol..

[31] Rodríguez-Rodríguez E., Sánchez-Prieto M., Olmedilla-Alonso B. (2020). Assessment of carotenoid concentrations in red peppers (*Capsicum annuum*) under domestic refrigeration for three weeks as determined by HPLC-DAD. Food Chem. X.

[32] Osório C., Machado S., Peixoto J., Bessada S., Pimentel F.B., Alves R.C., Oliveira M.B.P.P. (2020). Pigments content (Chlorophylls, fucoxanthin and phycobiliproteins) of different commercial dried algae. Separations.

[33] Wang X.F., Zhan C.H., Maoka T., Wada Y., Koyama Y. (2007). Fabrication of dye-sensitized solar cells using chlorophylls c1 and c2 and their oxidized forms c1′ and c2′ from *Undaria pinnatifida* (Wakame). Chem. Phys. Lett..

[34] Chen K., Ríos J.J., Pérez-Gálvez A., Roca M. (2017). Comprehensive chlorophyll composition in the main edible seaweeds. Food Chem..

[35] Schmid M., Stengel D.B. (2015). Intra-thallus differentiation of fatty acid and pigment profiles in some temperate Fucales and Laminariales. J. Phycol..

[36] Lewey S.A., Gorham J. (1984). Pigment composition and photosynthesis in *Sargassum muticum*. Mar. Biol..

[37] Schmid M., Guihéneuf F., Stengel D.B. (2017). Ecological and commercial implications of temporal and spatial variability in the composition of pigments and fatty acids in five Irish macroalgae. Mar. Biol..

[38] Roleda M.Y., Hanelt D., Kräbs G., Wiencke C. (2004). Morphology, growth, photosynthesis and pigments in *Laminaria ochroleuca* (Laminariales, Phaeophyta) under ultraviolet radiation. Phycologia.

[39] Rajauria G. (2019). In-vitro antioxidant properties of lipophilic antioxidant compounds from 3 brown seaweed. Antioxidants.

[40] Liu Z., Sun X., Sun X., Wang S., Xu Y. (2019). Fucoxanthin Isolated from *Undaria pinnatifida* can interact with *Escherichia coli* and *Lactobacilli* in the intestine and inhibit the growth of pathogenic bacteria. J. Ocean Univ. China.

[41] Rajauria G., Foley B., Abu-Ghannam N. (2017). Characterization of dietary fucoxanthin from *Himanthalia elongata* brown seaweed. Food Res. Int..

[42] Marinho G.S., Sørensen A.D.M., Safafar H., Pedersen A.H., Holdt S.L. (2019). Antioxidant content and activity of the seaweed *Saccharina latissima*: A seasonal perspective. J. Appl. Phycol..

[43] Bruhn A., Tørring D., Thomsen M., Canal-Vergés P., Nielsen M., Rasmussen M., Eybye K., Larsen M., Balsby T., Petersen J. (2016). Impact of environmental conditions on biomass yield, quality, and bio-mitigation capacity of *Saccharina latissima*. Aquac. Environ. Interact..

[44] Xiao X., Si X., Yuan Z., Xu X., Li G. (2012). Isolation of fucoxanthin from edible brown algae by microwave-assisted extraction coupled with high-speed countercurrent chromatography. J. Sep. Sci..

[45] López-Hernández J., de Quirós A.R.B. (2014). Evaluation of Bioactive Compounds in Seaweeds: A Review. Seaweeds: Agricultural Uses, Biological and Antioxidant Agents.

[46] Amorim-Carrilho K., Lage-Yusty M.A., López-Hernández J. (2014). Variation of bioactive compounds in dried seaweed *Himanthalia elongata* subjected to different culinary processes. CYTA J. Food.

[47] de Quirós A.R.B., Frecha-Ferreiro S., Vidal-Pérez A.M., López-Hernández J. (2010). Antioxidant compounds in edible brown seaweeds. Eur. Food Res. Technol..

[48] Kanda H., Kamo Y., Machmudah S., Wahyudiono, Goto M. (2014). Extraction of fucoxanthin from raw macroalgae excluding drying and cell wall disruption by liquefied dimethyl ether. Mar. Drugs.

[49] Kraan S. (2013). Pigments and Minor Compounds in Algae. Functional Ingredients from Algae for Foods and Nutraceuticals.

[50] Jacobsen C., Sørensen A.-D.M., Holdt S.L., Akoh C.C., Hermund D.B. (2019). Source, Extraction, Characterization, and Applications of Novel Antioxidants from Seaweed. Annu. Rev. Food Sci. Technol..

[51] Shannon E., Abu-Ghannam N. (2017). Optimisation of fucoxanthin extraction from Irish seaweeds by response surface methodology. J. Appl. Phycol..

[52] Vega J., Álvarez-Gómez F., Güenaga L., Figueroa F.L., Gómez-Pinchetti J.L. (2020). Antioxidant activity of extracts from marine macroalgae, wild-collected and cultivated, in an integrated multi-trophic aquaculture system. Aquaculture.

[53] Collén J., Davison I.R. (1999). Reactive oxygen metabolism in intertidal *Fucus* spp. (Phaeophyceae). J. Phycol..

[54] Rosa G.P., Barreto M.C., Seca A.M.L. (2019). Pharmacological effects of *Fucus spiralis* extracts and phycochemicals: A comprehensive review. Bot. Mar..

[55] Andersen G.S., Pedersen M.F., Nielsen S.L. (2013). Temperature acclimation and heat tolerance of photosynthesis in Norwegian *Saccharina latissima* (Laminariales, Phaeophyceae). J. Phycol..

[56] Nielsen M.M., Manns D., D’Este M., Krause-Jensen D., Rasmussen M.B., Larsen M.M., Alvarado-Morales M., Angelidaki I., Bruhn A. (2016). Variation in biochemical composition of *Saccharina latissima* and *Laminaria digitata* along an estuarine salinity gradient in inner Danish waters. Algal Res..

[57] Boderskov T., Schmedes P.S., Bruhn A., Rasmussen M.B., Nielsen M.M., Pedersen M.F. (2016). The effect of light and nutrient availability on growth, nitrogen, and pigment contents of *Saccharina latissima* (Phaeophyceae) grown in outdoor tanks, under natural variation of sunlight and temperature, during autumn and early winter in Denmark. J. Appl. Phycol..

[58] Vilg J.V., Nylund G.M., Werner T., Qvirist L., Mayers J.J., Pavia H., Undeland I., Albers E. (2015). Seasonal and spatial variation in biochemical composition of *Saccharina latissima* during a potential harvesting season for Western Sweden. Bot. Mar..

[59] Sousa G., Trifunovska M., Antunes M., Miranda I., Moldão M., Alves V., Vidrih R., Lopes P.A., Aparicio L., Neves M. (2021). Optimization of ultrasound-assisted extraction of bioactive compounds from *Pelvetia canaliculata* to sunflower oil. Foods.

[60] Martins M., Soares C., Figueiredo I., Sousa B., Torres A.C., Sousa-Pinto I., Veiga P., Rubal M., Fidalgo F. (2021). Fucoid macroalgae have distinct physiological mechanisms to face emersion and submersion periods in their southern limit of distribution. Plants.

[61] Yuan C.Y., Yang S., Wang Y., Cui Q.M. (2014). Effect of Temperature on the Growth and Biochemical Composition of Sargassum muticum. Proceedings of the Advanced Materials Research.

[62] Abdel Latef A.A.H., Srivastava A.K., Saber H., Alwaleed E.A., Tran L.S.P. (2017). *Sargassum muticum* and *Jania rubens* regulate amino acid metabolism to improve growth and alleviate salinity in chickpea. Sci. Rep..

[63] Kolb N., Vallorani L., Milanović N., Stocchi V. (2004). Evaluation of Marine Algae Wakame (*Undaria pinnatifida*) and Kombu (*Laminaria digitata japonica*) as Food Supplements. Food Technol. Biotechnol..

[64] Sugimura R., Suda M., Sho A., Takahashi T., Sashima T., Abe M., Hosokawa M., Miyashita K. (2012). Stability of fucoxanthin in dried *Undaria pinnatifida* (wakame) and baked products (scones) containing wakame powder. Food Sci. Technol. Res..

[65] Terasaki M., Kuramitsu Y., Kojoma M., Kim S.Y., Tanaka T., Maeda H., Miyashita K., Kawagoe C., Kohno S., Mutoh M. (2020). High fucoxanthin wakame (*Undaria pinnatifida*) prevents tumor microenvironment formation in an AOM/DSS mouse carcinogenic model. J. Funct. Foods.

[66] Garcia-Oliveira P., Carreira-Casais A., Caleja C., Pereira E., Calhelha R.C., Sokovic M., Simal-Gandara J., Ferreira I.C.F.R., Prieto M.A., Barros L. (2020). Macroalgae as an Alternative Source of Nutrients and Compounds with Bioactive Potential. Proceedings.

[67] Terasaki M., Narayan B., Kamogawa H., Nomura M., Stephen N.M., Kawagoe C., Hosokawa M., Miyashita K. (2012). Carotenoid profile of edible japanese seaweeds: An improved HPLC method for separation of major carotenoids. J. Aquat. Food Prod. Technol..

[68] Honda M., Kodama T., Kageyama H., Hibino T., Wahyudiono, Kanda H., Goto M. (2018). Enhanced Solubility and Reduced Crystallinity of Carotenoids, β-Carotene and Astaxanthin, by Z-Isomerization. Eur. J. Lipid Sci. Technol..

[69] Xie X., Lu X., Wang L., He L., Wang G. (2020). High light intensity increases the concentrations of β-carotene and zeaxanthin in marine red macroalgae. Algal Res..

[70] Cotas J., Leandro A., Monteiro P., Pacheco D., Figueirinha A., Gonçalves A.M.M., Jorge G., Pereira L. (2020). Seaweed Phenolics: From Extraction to Applications. Mar. Drugs.

[71] Cassani L., Gomez-Zavaglia A., Jimenez-Lopez C., Lourenço-Lopes C., Prieto M.A., Simal-Gandara J. (2020). Seaweed-based natural ingredients: Stability of phlorotannins during extraction, storage, passage through the gastrointestinal tract and potential incorporation into functional foods. Food Res. Int..

[72] Ramluckan K., Moodley K.G., Bux F. (2014). An evaluation of the efficacy of using selected solvents for the extraction of lipids from algal biomass by the soxhlet extraction method. Fuel.

[73] Martins M., Oliveira R., Coutinho J.A.P., Faustino M.A.F., Neves M.G.P.M.S., Pinto D.C.G.A., Ventura S.P.M. (2021). Recovery of pigments from *Ulva rigida*. Sep. Purif. Technol..

[74] Cvitković D., Dragović-Uzelac V., Dobrinčić A., Čož-Rakovac R., Balbino S. (2021). The effect of solvent and extraction method on the recovery of lipid fraction from Adriatic Sea macroalgae. Algal Res..

[75] Martins M., Mesquita L.M.D.S., Vaz B.M.C., Dias A.C.R.V., Torres-Acosta M.A., Quéguineur B., Coutinho J.A.P., Ventura S.P.M. (2021). Extraction and Fractionation of Pigments from *Saccharina latissima* (Linnaeus, 2006) Using an Ionic Liquid + Oil + Water System. ACS Sustain. Chem. Eng..

[76] Obluchinskaya E.D., Pozharitskaya O.N., Zakharova L.V., Daurtseva A.V., Flisyuk E.V., Shikov A.N. (2021). Efficacy of natural deep eutectic solvents for extraction of hydrophilic and lipophilic compounds from *Fucus vesiculosus*. Molecules.

[77] Hallerud C.B. (2014). Pigment Composition of Macroalgae from a Norwegian Kelp Forest. https://ntnuopen.ntnu.no/ntnu-xmlui/handle/11250/238838.

[78] MarLIN: The Marine Life Information Network. for Britain & Ireland. https://www.marlin.ac.uk/.

